# In Vivo Characterization of Mucin and Amyloid Deposits in Primary Basal Cell Carcinoma through Reflectance Confocal Microscopy: A Correlation with Histopathology

**DOI:** 10.3390/diagnostics13030422

**Published:** 2023-01-24

**Authors:** Mihai Lupu, Ana Maria Malciu, Vlad Mihai Voiculescu

**Affiliations:** 1 Department of Dermatology, Victoria Medical Center, 030442 Bucharest, Romania; 2 Department of Dermatology, “Carol Davila” University of Medicine and Pharmacy, 050474 Bucharest, Romania; 3 Department of Dermatology and Allergology, Elias Emergency University Hospital, 011461 Bucharest, Romania

**Keywords:** basal cell carcinoma, reflectance confocal microscopy, amyloid, mucin, colloidal iron, cytokeratin 34betaE12, non-melanoma skin cancer

## Abstract

Basal cell carcinoma (BCC) is the most common keratinocyte carcinoma and the most prevalent skin cancer in humans, worldwide. BCC is histologically characterized by the proliferation of basaloid cells, arranged in globular masses of varying size, often separated from the surrounding stroma by optically empty spaces. Although attributed to tumor retraction during tissue processing for the preparation of pathology slides, these spaces are also seen on cryostat sections. The aim of this study is to in vivo characterize amyloid and mucin deposits in primary BCC lesions through RCM, followed by histopathological correlation. We included twenty-two consecutive subjects totaling thirty-one primary BCCs. Each lesion underwent the same evaluation protocol which included: clinical and dermoscopic images, RCM imaging, excisional biopsy under local anesthesia, and histopathological examination (colloidal iron and cytokeratin 34betaE12 stains). Hypo-reflective peritumoral clefts and hyper-reflective globules were measured on RCM images and compared to mucin and amyloid deposits seen on histology slides. The mean differences between RCM and histology measurements in both mucin and amyloid were not statistically significant. There were medium and strong correlations between RCM and histology regarding mucin and amyloid deposits, respectively.

## 1. Introduction

Basal cell carcinoma (BCC) is the most frequently occurring keratinocyte carcinoma and the most common skin cancer in humans, worldwide. BCC can manifest as multiple subtypes, including superficial, nodular, morpheaform, micro-nodular, infiltrative, as well as pigmented variants.

BCCs are histologically characterized by the proliferation of basaloid cells, arranged in globular masses of varying size, sometimes appended to the epidermis, sometimes invading the underlying dermis. Tumor nodules display peripheral palisading of cells, are surrounded by a distinctive stroma and are often separated from it by empty spaces, or clefts. 

These optically empty clefts have been seen as an artifact, generated through tumor retraction during tissue processing for the preparation of pathology slides [1]. This phenomenon has been, in the past, linked to the reduced synthesis of a basal membrane protein by basaloid BCC cells, rendering tumor nodules less adhesive to the surrounding tissue [2]. Nevertheless, the clefts surrounding BCC tumor islands also show up in cryostat sections [1], suggesting the contribution of additional factors to this finding.

Less-aggressive BCC subtypes, such as nodular BCC, have been reported to be up to 85% positive for amyloid deposition [3].

Since the introduction of in vivo reflectance confocal microscopy (RCM) as a non-invasive imaging tool in dermatology, numerous studies have described multiple criteria for BCC diagnosis with a good correlation to conventional histopathological findings [4,5]. 

RCM allows for the real-time, in vivo evaluation of the skin at quasi-histological resolution in horizontal, grayscale, and optical sections. A recently published multicenter study assessed the sensitivity and specificity of RCM for the diagnosis of primary BCC and found them to be 97.1% and 78.95%, respectively [6]. The same study found that peritumoral collagen bundles and increased vascularization are predictive of nodular BCC, cords connected to the epidermis predict superficial BCC, and hyporeflective silhouettes characterize aggressive BCC (micronodular, infiltrative, and basosquamous subtypes). Other criteria such as keratinocyte atypia, epidermal streaming, ulceration, and inflammation were found to be associated with all BCC histopathological subtypes [6].

Additionally, narrow, hypo-reflective, cleft-like spaces separating tumor islands from the surrounding dermis have been previously described [7,8] and correlated with the optically empty clefts seen in paraffin-embedded and cryostat sections of BCC. It is now known that these clefts exist in vivo and appear through the peritumoral deposition of mucin [8]. 

RCM descriptions of primary cutaneous amyloidosis have been published [9,10], peritumoral mucin on RCM has been histopathologically verified in BCC [8], and both amyloid and mucin have been described through RCM-optical coherence tomography (RCM-OCT) [11]. 

In this paper, we report the in vivo characterization of amyloid and mucin in primary BCC lesions through in vivo RCM, followed by histopathological correlation.

## 2. Materials and Methods

### 2.1. Study Population

Twenty-two consecutive Caucasian subjects (Fitzpatrick phototypes II and III), aged between 32 and 90 years, with a clinical diagnosis of BCC were enrolled. Five of the subjects were smokers (4 females and one male), but none of them had a history of significant skin diseases. The study was conducted in accordance with the Declaration of Helsinki Principles, and informed consent was obtained from each patient prior to enrollment. The study protocol was approved by the Ethics Committee of the “Carol Davila” University of Medicine and Pharmacy Bucharest (Project Number 185/26.12.2018).

Clinical examination was performed according to the European guidelines for the inspection of skin cancer [12] and included dermoscopy.

### 2.2. Data Acquisition

A commercially available reflectance confocal microscope (VivaScope 1500; Caliber ID, Henrietta, NY, USA; MAVIG GmbH, München, Germany) was used to acquire confocal images. A detailed description of the technique and device can be found elsewhere [6]. First, macroscopic and dermoscopic images were acquired using the VivaCam (Caliber ID, Henrietta, NY, USA; MAVIG GmbH, München, Germany) module of the confocal microscope. Then, RCM imaging was performed according to a prespecified protocol: 6x6 mm horizontal composite images (mosaics) were captured at three depth points, the stratum spinosum, the dermal-epidermal junction (DEJ), and the upper dermis, starting in the center of the lesion. Also, consecutive single confocal images were captured at different depths (stacks), starting at the stratum corneum, in 5 μm steps, down to a maximum depth of approximately 250 μm. Furthermore, three to six individual images were obtained during the imaging process.

RCM images were evaluated for: the presence of BCC criteria [6], mucin (presenting as hyporeflective areas located intra- and/or peritumorally), and amyloid (presenting as amorphous, hyper-reflective, relatively homogenous globules located intra- and/or peri-tumorally) [8,9].

Dermoscopy and confocal imaging acquisition and evaluation were performed by an investigator (ML) with over 6 years of experience with in vivo RCM imaging.

Tissue specimens were obtained through excisional biopsy under local anesthesia from 31 skin lesions suggestive of BCC upon clinical, dermoscopic, and RCM evaluation. After fixation in formalin, 4-μm-thick, vertical sections were made from paraffin-embedded blocks and stained with Hematoxylin–Eosin (H&E), Colloidal Iron (CI, for mucin), and Cytokeratin 34betaE12 (CK34βE12, for amyloid).

Histopathological examination in all cases was performed by the same highly experienced dermatopathologist, which was blinded to clinical, dermoscopical, and RCM data.

The size and distribution of hyper-reflective globules and the width of peritumoral clefts on RCM images were measured using the open-source software FIJI (ImageJ, NIH). For each of the two types of structures, three random measurements were performed on representative images in each lesion. The width of the dark hyporeflective spaces was measured as the distance between two points situated on either side of the cleft. Hyper-reflective globule size was considered as its largest measurable diameter. All measurements were expressed in microns (μm). The thickness of peritumoral mucin and size of amyloid deposits on histological slides were measured using the same open-source software, FIJI (ImageJ, NIH), on calibrated photomicrographs, and expressed in microns (μm).

### 2.3. Statistical Analysis

Statistical analysis included calculation of the size of peritumoral clefts and hyper-reflective globule size for both RCM and histopathology, expressed as mean and standard deviation. Next, the means of RCM and histopathology measurements for both mucin and amyloid deposits were compared using a paired-sample t-test. Finally, the calculation of the Pearson correlation index between RCM measurements and histological measurements was performed. All statistical analyses were conducted with the SPSS v22 software package (IBM).

## 3. Results

We included twenty-two subjects (7 males, 15 females) with a mean age of 69 ± 13.58 (range 32–90) years which totaled thirty-one BCCs (4 superficial, 22 nodular, 2 nodulocystic, and 3 noduloinfiltrative). Lesions were predominantly located on the head and neck (20/31), followed by the trunk (6/31), and upper (4/31) and lower extremities (1/31).

**Figure 1:**
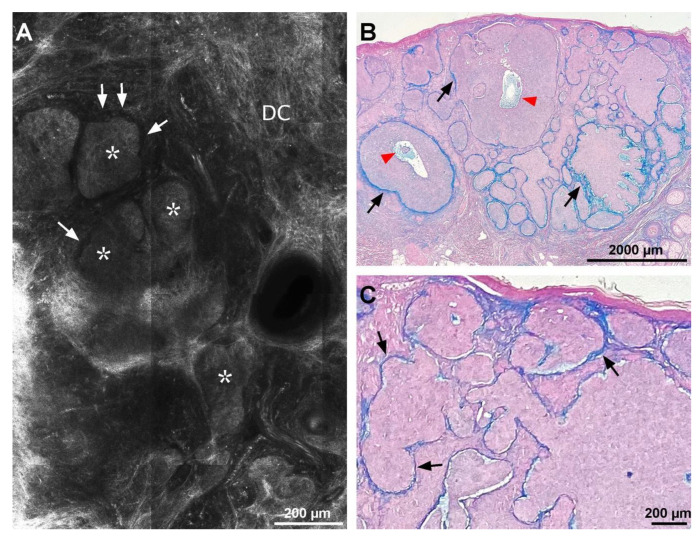
Correlation of reflectance confocal microscopy (RCM) image and colloidal iron-stained histological aspect of a nodular basal cell carcinoma (BCC). (**A**) RCM mosaic showing basaloid tumor nodules (white asterisks) displaying peripheral palisading, separated from dermal collagen (DC) by hyporeflective peritumoral spaces also known as clefts (white arrows). (**B**) Photomicrograph showing blue mucin deposits around, in between (black arrows), and inside (red arrowheads) single BCC tumor nodules (colloidal iron stain). (**C**) Histology corresponding to panel (**A**) showing BCC tumor islands separated from the adjacent dermis by mucin staining blue (black arrows) (colloidal iron stain). RCM and histopathology images belong to the same case.

RCM imaging revealed peritumoral clefting in all lesions. The clefts appeared as dark, hypo-reflective spaces between tumor islands and the surrounding stroma (Figure 1A). Their width varied between lesions, and also within each lesion, from 11.26 to 23.43 μm (mean 16.57 ± 3.49 μm) (Table 1).

**Figure 2:**
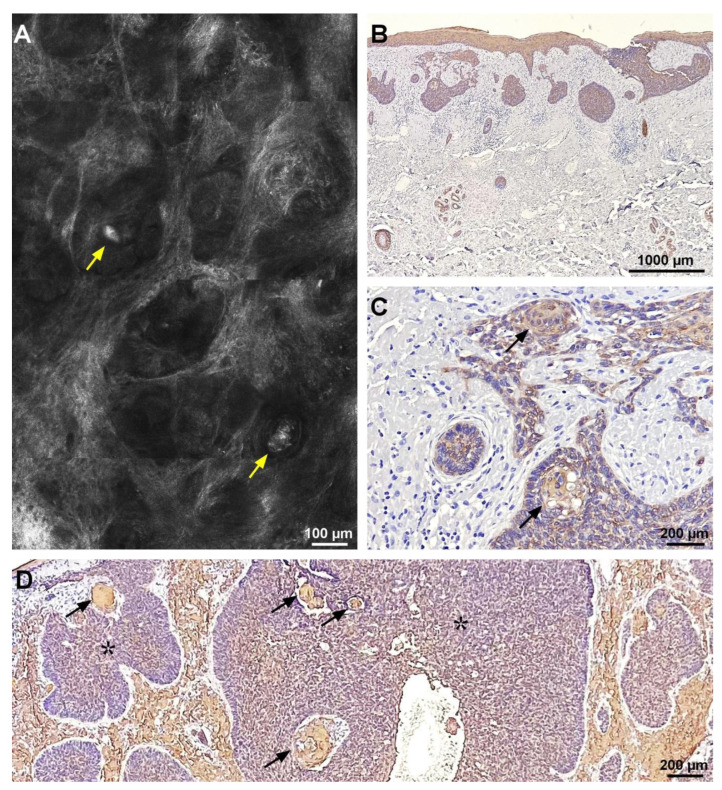
Correlation of reflectance confocal microscopy (RCM) image and CK34βE12 histological aspect of a nodular basal cell carcinoma (BCC). (**A**) RCM mosaic showing BCC tumor nodules with peripheral palisading and hyper-reflective globular structures within them (yellow arrows). (**B**) Histological image showing amyloid deposits stained in light brown (CK34βE12 stain). (**C**) Histology corresponding to panel (**A**) showing intra-tumoral amyloid deposits staining light brown (black arrows) (CK34βE12 stain). (**D**) Histological image displaying light brown, globular amyloid deposits (black arrows) inside BCC tumor islands (black asterisks) (CK34βE12 stain). RCM and histopathology images belong to the same case.

Hyper-reflective globules were distributed peritumorally or within hyporeflective areas (Figure 2A) and could be observed in 28 of the 31 lesions. The mean hyper-reflective globule size also varied between lesions, and also within each lesion, from 11.72 to 134.13 μm (mean 55.77 ± 26.77 μm) (Table 1).

Histopathological examination confirmed the diagnosis of basal cell carcinoma in all 31 lesions included in the study. Colloidal iron staining showed the presence of peritumoral mucin of variable thickness as an amorphous, blue staining substance, in all lesions. Mucin was present around the tumor islands at the level of the dermis but was also seen within the tumor masses (Figure 1B). The mean thickness of peritumoral mucin deposits varied between 10.73–32.10 μm (mean, 17.15 ± 4.48 μm) (Table 1). CK34βE12 staining showed the presence of amyloid as homogenous brown globules in thirty of the 31 lesions. Amyloid deposits could not be identified on histology in one case of nodulo-infiltrative BCC. Amyloid deposits were found both peritumorally, but also within tumor islands (Figure 2D). The size of amyloid globules varied between 12.56–120.32 μm (mean, 59.9 ± 27.37 μm) (Table 1).

The mean difference between RCM cleft width and histologically measured mucin deposits was −0.576 µm (95%CI [−1.801–0.647]), and was not statistically significant (*p* = 0.344). We found a mean difference of −5.41 µm (95%CI [−10.98–0.159]) between RCM hyper-reflective globule size and histologically measured amyloid deposits (in 28/31 cases), which was also not significant (*p* = 0.056).

**Figure 3:**
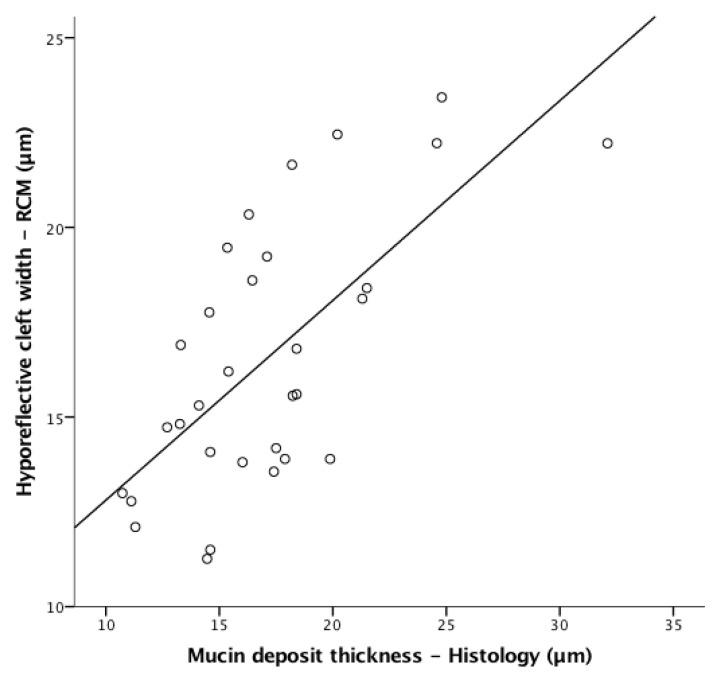
Scatterplot showing the correlation for mucin deposits seen on reflectance confocal microscopy (RCM) and histology. Linear correlation between cleft-like hyporeflective spaces seen on RCM examination and mucin deposits on colloidal iron-stained tissue sections. The range of measurements is fairly wide, resulting in a Pearson r = 0.675 (*p* < 0.001).

A linear correlation was present between the hypo-reflective clefts observed by RCM and the presence of mucin on histopathologic examination (Pearson r = 0.675, *p* < 0.001; Figure 3). 

**Figure 4:**
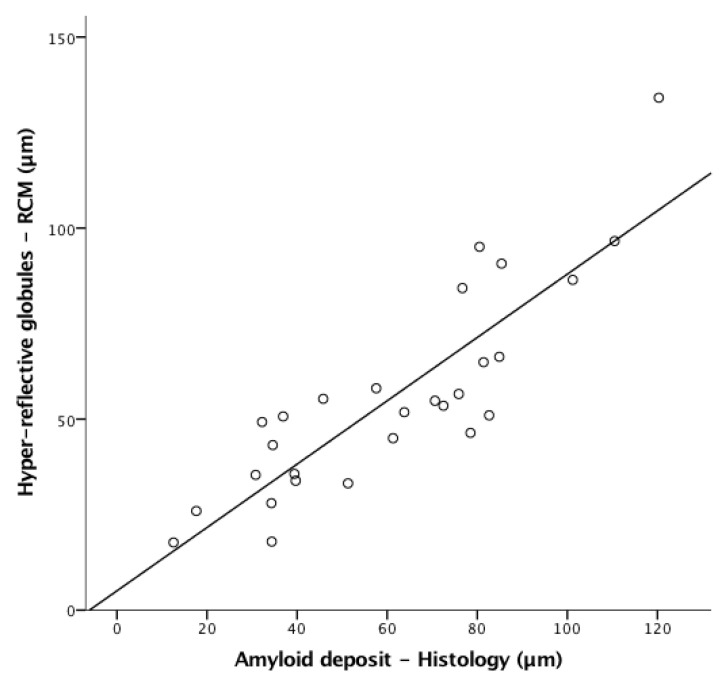
Scatterplot showing the correlation for amyloid deposits seen on reflectance confocal microscopy RCM and histology. Linear correlation between hyper-reflective globules seen on RCM examination and amyloid deposits on CK34βE12-stained tissue sections. The correlation was strong, resulting in a Pearson correlation of r = 0.862 (*p* < 0.001).

A strong correlation was also found between hyper-reflective globule mean size as measured by in vivo RCM and amyloid deposit mean size on histology (Pearson correlation r = 0.862, *p* < 0.001; Figure 4).

## 4. Discussion

To facilitate diagnosis and shorten the delay between diagnosis and treatment, several diagnostic RCM criteria have been defined for BCC, features that correlate well with established histological ones [6]. 

The presence of narrow, hypo-reflective, cleft-like spaces has long been observed on RCM images of BCC. These peritumoral spaces are also a common finding in histopathological sections and have been considered an artifact, caused by the shrinking of BCC nodules induced by tissue fixation and dehydration during processing for conventional histology. The separation of BCC nodules from the stroma would also be facilitated by the reduced production of basement membrane antigens by BCC cells [2]. It was Ulrich et al. who pioneered that due to their presence on RCM imagery and cryostat sections these clefts are not (solely) due to tissue processing for routine pathology, and that they represent, in fact, the deposition of peritumoral mucin [8]. Even though this research shows mucin’s role in the appearance of these hypo-reflective spaces, there are instances when these peritumoral spaces are, in fact, proper halo spaces.

This study aimed to describe mucin and amyloid deposits in primary BCC lesions through in vivo RCM, followed by precise correlation on pathology slides stained with colloidal iron and CK34βE12.

In their study, Ulrich et al. [8] recognize several limitations that would contribute to the rather weak correlation between peritumoral cleft width on RCM and histopathology. Namely, the uneven distribution of mucin around tumor nodules, the discontinuity of peritumoral clefts on RCM images, the different orientations of RCM and histology sections, and last but not least the degree of tissue contraction due to processing which inevitably occurs and contributes to peritumoral cleft shrinking.

To mitigate some of these limitations, we used RCM image stacks to fully evaluate the peritumoral clefts. Interestingly, in some lesions examined in this study, histologically measured distances were larger (albeit, not statistically significant) compared to those measured on RCM images. This finding could be attributed to the use of different stains for mucin (colloidal iron) and amyloid (CK34βE12) compared to previous studies which used toluidine blue and Congo red, respectively [8,11].

The histopathological BCC subtypes included in this study were superficial, nodular, nodulocystic, and noduloinfiltrative. Our results showed moderate and very good correlations between RCM and histopathology in regard to hypo-reflective peritumoral clefting and hyper-reflective globules, respectively. The width of peritumoral clefts on RCM and of the peritumoral mucin on histopathology correlated fairly well, thus enforcing the finding that the clefts visualized by RCM are a result of mucin deposition. The mean size of hyper-reflective globules as measured by RCM also correlated well with the amyloid deposits measured on histological photomicrographs, suggesting that the structures visible on RCM are indeed amyloid deposits.

Amyloid and mucin deposition are commonly observed in less-aggressive BCC subtypes, such as nodular BCC (up to 85% positive for amyloid) [3]. Our findings confirmed the presence of amyloid through in vivo RCM in 28 of the 31 lesions, all of which were non-aggressive. In two of the three remaining lesions amyloid deposits were found deeper inside the dermis, thus being out of reach of confocal examination. In one of the nodulo-infiltrative BCCs included in this study amyloid deposits could not be identified by means of histology. 

The strong correlation between structures visible on RCM images and histopathology slides suggests that the non-invasive visualization of pronounced amyloid and mucin features may be clinically relevant for the diagnosis of less-aggressive BCCs, which in turn can streamline the management of these tumors.

## Figures and Tables

**Table 1:** Peritumoral hyporeflective cleft width and hyper-reflective globule sizes obtained by three random measurements in histological samples and RCM images of the lesions.

Structure	Mean Value (μm)
Hypo-reflective peritumoral spaces (RCM)	16.57 ± 3.49
Peritumoral mucin deposits (Histology)	17.15 ± 4.48
Hyper-reflective globules (RCM)	55.77 ± 26.77
Amyloid deposits (Histology)	59.9 ± 27.37

RCM, reflectance confocal microscopy.

## Data Availability

The data presented in this study are available on request from the corresponding author.
